# Epidermal Stem Cells Are Defined by Global Histone Modifications that Are Altered by Myc-Induced Differentiation

**DOI:** 10.1371/journal.pone.0000763

**Published:** 2007-08-22

**Authors:** Michaela Frye, Amanda G. Fisher, Fiona M. Watt

**Affiliations:** 1 Wellcome Trust Centre for Stem Cell Research, University of Cambridge, Cambridge, United Kingdom; 2 Lymphocyte Development Group, Imperial College School of Medicine, London, United Kingdom; 3 Cancer Research UK Cambridge Research Institute, University of Cambridge, Cambridge, United Kingdom; Wellcome Trust Sanger Institute, United Kingdom

## Abstract

Activation of Myc induces epidermal stem cells to exit their niche and differentiate into sebocytes and interfollicular epidermis, a process that is associated with widespread changes in gene transcription. We have identified chromatin modifications that are characteristic of epidermal stem cells and investigated the effects of Myc activation. Quiescent stem cells in the interfollicular epidermis and the hair follicle bulge had high levels of tri-methylated histone H3 at lysine 9 and H4 at lysine 20. Chromatin in both stem cell populations was hypoacteylated at histone H4 and lacked mono-methylation of histone H4 at lysine 20. Myc-induced exit from the stem cell niche correlated with increased acetylation at histone H4 and transiently increased mono-methylation at lysine 20. The latter was replaced by epigenetic modifications that are largely associated with chromatin silencing: di-methylation at histone H3 lysine 9 and histone H4 lysine 20. These modifications correlated with changes in the specific histone methyltransferases Set8 and Ash-1. The Myc-induced switch from mono- to di-methylated H4K20 required HDAC activity and was blocked by the HDAC inhibitor trichostatin A (TSA). TSA treatment induced a similar epidermal phenotype to activation of Myc, and activation of Myc in the presence of TSA resulted in massive stimulation of terminal differentiation. We conclude that Myc-induced chromatin modifications play a major role in Myc-induced exit from the stem cell compartment.

## Introduction

Many histone modifications, including acetylation, phosphorylation, ubiquitination, sumoylation, and methylation, are known to regulate chromatin structure and gene expression [1], [2]. This is illustrated by modification of histone H3. When a gene is transcriptionally active histone H3 is acetylated at lysines 9 and 14 and di- or tri-methylated at lysine 4. Conversely, in inactive chromatin histone H3 is di- or tri-methylated at lysine 9 or 27 [2]. Epigenetic modifications are set by cell-type specific transcriptional regulators and chromatin remodelling enzymes [3].

There is growing evidence that specific chromatin modifications distinguish stem and differentiated cells in a wide range of tissues. In Drosophila, germ line and somatic stem cell self-renewal are controlled by the chromatin remodelling factors ISWI and DOM, respectively [4]. In neural stem cells epigenetic marks are believed to be the main intrinsic factor regulating self-renewal and differentiation [5]. In the haematopoietic system quiescent B lymphocytes are characterised by global hypomethylation at histone H3 [6]. Under-representation of repressive histone marks could be indicative of epigenetic plasticity in stem cells [7].

Mammalian epidermis provides an excellent model in which to analyse the state and significance of chromatin modifications in stem cells and their progeny. There are two reasons for this. The first is that the location of at least two stem cell pools, in the hair follicle bulge and in human interfollicular epidermis, is well established [8], [9], [10]. The second is that activation of the transcription factor Myc triggers exit from the epidermal stem cell compartment and induces differentiation along the sebaceous and interfollicular epidermal lineages [11], [12]. Recent studies suggest that Myc acts as a widespread regulator of gene transcription [13], [14], and both activation and repression of gene expression contribute to the Myc-induced epidermal phenotype [15], [16], [17]. The biochemical mechanism of Myc-mediated transactivation has revealed a wide range of effects on chromatin and basal transcription [18]. Myc proteins are required for the widespread maintenance of active chromatin [19].We therefore set out to investigate whether adult epidermal stem cells have common epigenetic modifications and how these change in response to Myc activation.

## Results

### Histone marks in human epidermis

**Figure 1:**
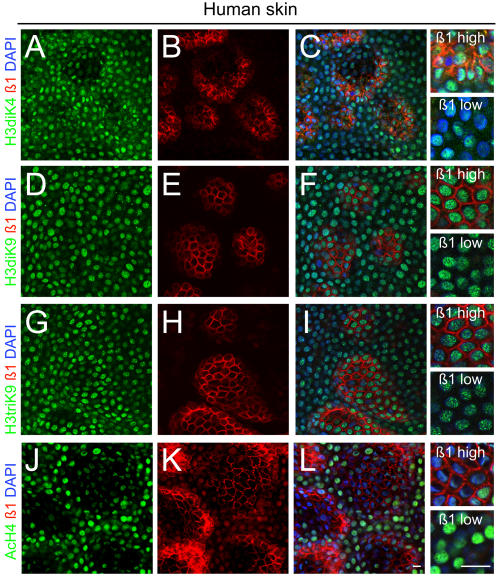
Histone modifications in basal layer of human interfollicular epidermis. Double label immunofluorescence staining of whole mounts with antibodies to ß1 integrins (red) and (green) H3diK4 (A–C), H3diK9 (D–F), H3triK9 (G–I) and H4Ac (J–L), with DAPI nuclear counterstain (blue). C, F, I, L show merged images (left hand panels) and higher magnification views (right hand panels). Scale bars: 20 µm.

We began by investigating whether stem cells in human interfollicular epidermis were characterised by specific histone modifications. We prepared epidermal whole mounts [9], [20] and labelled them with antibodies specific for histone H3 methylation at lysines 4 (H3diK4) or 9 (H3diK9, H3triK9) and an antibody that detects acetylation of H4 (H4Ac) (Figure 1).

Human interfollicular epidermal stem cells express high levels of ß1 integrins and are arranged in clusters in the epidermal basal layer, surrounded by their progeny, transit amplifying (TA) cells, and cells that have initiated terminal differentiation. Double labelling for β1 integrins and antibodies to modified histones revealed that although levels of H3diK4 or H3di, triK9 methylation varied, there was no correlation with high or low expression of ß1 integrins (Fig. 1A–I). In contrast, cells that expressed high levels of ß1 integrins had low levels of H4 acetylation (Fig. 1J–L). Therefore low levels of H4 acetylation are a marker of human interfollicular epidermal stem cells.

### Histone marks in the mouse hair follicle bulge

**Figure 2:**
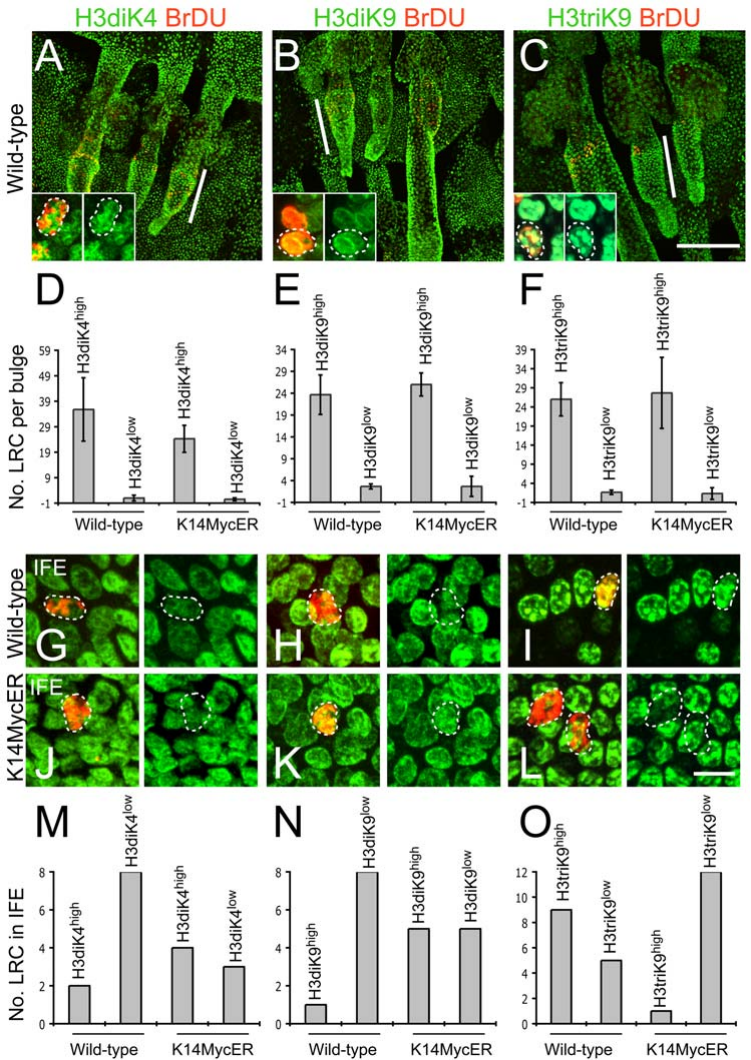
Variation in histone H3 methylation in mouse epidermis and effects of Myc activation. Immunofluorescence staining for LRC (red) and (green) H3diK4 (A, G, J), H3diK9 (B, H, K) and H3triK9 (C, I, L) in whole mounts of wild type (A–C, G–I) and 4OHT treated K14MycER (J–L) epidermis. Position of hair follicle bulge is marked by line in A–C. Inserts in A–C show higher magnification views of bulge LRC. G–L show LRC in interfollicular epidermis. (D–F, M–O) Quantitation (±S.D.) of number of LRC per bulge (D–F) and interfollicular epidermis (M–O) that had high or low levels of staining (see Fig. S1) with antibodies to the H3 modifications indicated. Scale bars: 100 µm (A–C), 20 µm (G–L).

In mouse epidermis the best characterised pool of stem cells is not in the interfollicular epidermis but in a region of the hair follicle known as the bulge [8]. One characteristic of bulge stem cells is that if they incorporate BrdU in neonatal epidermis they retain the label in adulthood; such cells are known as label retaining cells (LRC) [21], [22]. We therefore examined the state of histone modifications in label retaining cells (LRC) in whole mounts of mouse tail epidermis (Fig. 2A–F) [22]. Antibodies to H3diK4, H3diK9 and H3triK9 gave uniform labelling along the outer root sheath (ORS) of the hair follicle, and most LRC, like the rest of the bulge cells, were strongly labelled (Fig. 2A–C; line; insert). To quantify these results we counted the total number of LRC per bulge and scored each cell as strongly (high) or weakly (low) labelled (Fig. 2D–F, Fig. S1).

Whereas levels of histone methylation appeared to be uniform in bulge cells, we detected heterogeneity amongst cells of the interfollicular epidermis (IFE) (Figure 2G–I). The majority of BrdU positive LRC in wild-type interfollicular epidermis were low in H3diK4 or H3diK9 methylation (Fig. 2 G,H and M,N), but high in H3triK9 (Fig. 2I,O).

We conclude that LRC in the bulge and the IFE represent distinct cell populations when characterised by the level of H3 methylation.

### Effect of Myc activation on epidermal histone marks

Epidermal stem cells can be stimulated to exit the stem cell compartment by activation of the transcription factor Myc [23]. In response to Myc, stem cells move into the TA compartment, where they undergo several rounds of division and then differentiate along the lineages of the interfollicular epidermis and sebaceous gland [11], [12], [15], [17]. To investigate the consequences of overexpressing Myc on histone modifications in epidermal cells, we prepared whole mounts of tail epidermis from K14MycER mice that had been treated with 4-hydroxy-tamoxifen (4OHT). The keratin 14 promoter drives transgene expression in stem and transit amplifying cells in all regions of the epidermis. MycER is a chimeric protein in which the C-terminus of Myc is fused to a mutant oestrogen receptor; thus Myc is only activated when cells are treated with 4OHT [11], [12].

As reported previously [22], activation of Myc did not alter the number or location of LRC in the bulge or IFE (Fig. 2D–F, M–O and data not shown). In the bulge, the level of methylation of H3diK4 and H3di, triK9 was similar in 4OHT treated K14MycER mice and wild-type littermates (Fig. 2D–F). However, the IFE differed from the bulge in its response to Myc. We still detected LRC with low levels of H3diK4 and H3diK9 methylation (Fig. 2M,N), but the proportion of cells with high levels of these modifications was increased compared to wild-type control LRC (Fig. 2M,N).

The most dramatic effect of Myc was on H3triK9 methylation of interfollicular epidermal LRC. The majority of LRC in Myc activated epidermis were low in H3triK9 methylation, whereas in controls most of the LRC had high levels (Fig. 2O). This suggests that Myc increased H3diK9 methylation at expense of H3triK9 methylation.

### Low levels of H4 acetylation are a mark of label retaining cells in mouse epidermis that is regulated by Myc

**Figure 3:**
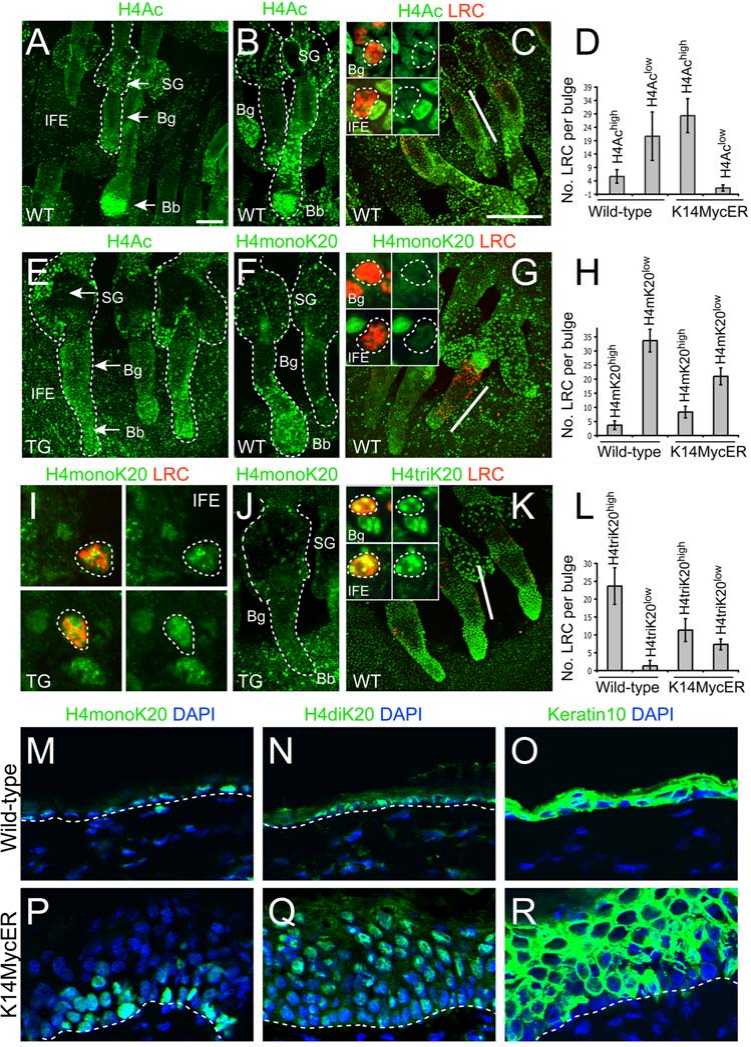
Variation in histone H4 acetylation and methylation in mouse epidermis and effects of Myc activation. (A–C, E–G, I–K) Immunofluorescence staining of whole mounts of wild-type (A–C, F, G, K) and 4OHT treated K14MycER transgenic (E,I, J) epidermis with antibodies to (green) H4Ac (A–C, E), H4monoK20 (F, G, I, J) and H4triK20 (K). Double labelling for LRC (red) is shown in C, G, I, K. I and inserts in C, G, K show high magnification views of LRC. IFE: interfollicular epidermis; sebaceous gland (SG), bulge (Bg), bulb (Bb) are marked with arrows in A, E. Hair follicles and sebaceous glands are marked with dashed lines in A, B, E, F, J; LRC are marked with dashed lines in C, G, I, K; position of bulge is marked with line in C, G, K. D, H, L show quantitation (±S.D.) of number of LRC per bulge with high or low levels of the H4 modifications indicated. M–R: immunofluorescence labelling (green) with DAPI counterstain (blue) of wild-type (M–O) and 4OHT treated K14MycER (P–R) dorsal interfollicular epidermis using antibodies to H4monoK20 (M, P), H4diK20 (N, Q) and keratin 10 (O, R). Dashed lines indicate position of basement membrane. Scale bar: 50 µm

Since low H4 acetylation was common in stem cells in human interfollicular epidermis (Fig. 1), we examined whether this was also the case in mouse epidermis (Fig. 3A–D). The entire bulge had much lower levels of histone acetylation than the rest of the hair follicle (Fig. 3A,B; Bg). Double labelling for BrdU and H4Ac demonstrated that LRC in both the bulge and interfollicular epidermis of wild-type mice had low acetylation of H4 (Fig. 3C,D; insert). We conclude that in human and mouse interfollicular epidermis and in the mouse hair follicle bulge, stem cells are characterised by low levels of histone H4 acetylation.

Acetylation of histone H3 and H4 is a chromatin modification known to be mechanistically associated to Myc-induced transactivation. Myc regulates gene transcription by recruiting the histone acetyltransferases GCN5 and TIP60, and by directly up-regulating GCN5 [19], [24], [25]. Therefore it was not surprising that in 4OHT treated K14MycER epidermis there was increased H4 acetylation of bulge LRC (Fig. 3D). Indeed, on activation of Myc the bulge could no longer be distinguished on the basis of low H4 acetylation (Fig. 3A,E; Bg), and acetylation was also increased in the interfollicular epidermis (Fig. 3A,E; IFE).

We next asked whether H4 acetylation was correlated with methylation at lysine 20 (K20). Mono-methylation of histone H4 at lysine 20 (H4monoK20) is increased in the promoter and coding regions of active genes and correlates with histone hyperacetylation [26]. In the epidermis of wild-type mice, levels of H4monoK20 correlated with acetylation of H4, being low in the bulge (Bg) and high in other regions of the epidermis, particularly the hair follicle bulb (Bb) (Fig. 3 B,F).

The increase in H4 acetylation resulting from activation of Myc correlated with increased levels of H4monoK20 in bulge and interfollicular epidermal LRC (Fig. 3G–I). However, the increase was modest and did not occur throughout the whole epidermis (Fig. 3J), leading us to speculate that H4monoK20 might accumulate only transiently and be replaced with another modification, such as H4di- or triK20. Since the number of LRC with high levels of H4triK20 was decreased in 4OHT treated K14MycER epidermis (Fig. 3K,L), we speculated that H4monoK20 might be replaced by H4diK20.

### Myc-induced switch from H4monoK20 to H4diK20 depends on HDAC activity

To demonstrate that H4diK20 was up-regulated in vivo, we treated K14MycER and wild-type control mice for 6 days with 4OHT. H4diK20 was dramatically increased in K14MycER mice compared to the controls (Fig. 3N,Q). Whereas nuclei high in H4monoK20 were localised to the basal layer of the IFE in control and transgenic mice (Fig. 3M,P), cells with high levels of H4diK20 were found in the differentiating, keratin 10 positive, suprabasal layers of K14MycER mice (Fig. 3N–R).

**Figure 4:**
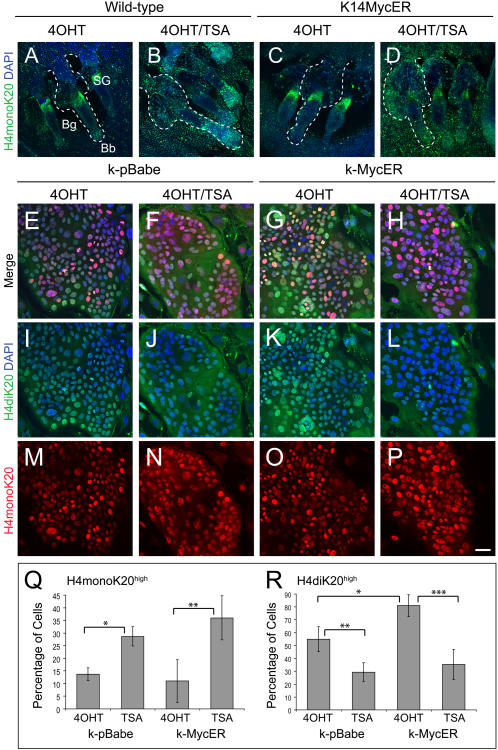
Effects of TSA treatment and Myc activation on histone H4 modification. (A–D) whole mounts of wild-type (A, B) and K14MycER (C, D) epidermis treated with 4OHT alone (A, C) or in combination with TSA (B, D), labelled with antibody to H4monoK20 (green) and counterstained with DAPI (blue). Hair follicles and sebaceous glands (SG) are outlined with dashed lines. Bg: bulge; Bb: bulb (A–D). (E–P) Double immunofluorescence labelling of cultured human keratinocytes transduced with empty vector (k-pBabe; E, F, I, J, M, N) or MycER (k-MycER; G, H, K, L, O, P) and treated for 24 h with 4OHT alone (E, I, M, G, K, O) or 4OHT+TSA (F, J, N, H, L, P). Cells were stained with antibodies to H4monoK20 (red) and H4diK20 (green) with DAPI counterstain (blue). E–H are merged images of the panels below. (Q,R) Quantitation (±S.D.) of % cells per colony with high levels of H4monoK20 (Q) or H4diK20 (R). Cells were stimulated with 4OHT alone (4OHT) or 4OHT and TSA (TSA). P values (t-test) are *0.0046, **0.0505 (Q) and *0.0545, **0.0211, ***0.0192 (R). Scale bar: 100 µm (A–D), 20 µm (E–P).

The re-organisation of constitutive heterochromatin from H4monoK20 to H4diK20 requires HDAC activity [27]. If Myc replaces H4monoK20 with H4diK20, the process should be blocked with the HDAC inhibitor, trichostatin A (TSA) [28]. When we treated wild-type mouse epidermis with TSA, increased numbers of cells with H4monoK20 were found in the IFE, hair follicles and sebaceous glands (Fig. 4A,B). The effect was even more pronounced when TSA was combined with Myc activation (Fig. 4C,D).

To examine the mechanism by which Myc induced the switch from H4monoK20 to H4diK20, cultured primary human keratinocytes transduced with a MycER retroviral vector or an empty vector control (pBabe) were labelled with antibodies to the different states of H4 methylation (Fig. 4E–P). The number of cells with high levels of H4diK20 increased by approximately 25% on activation of Myc (Fig. 4I,K,R), an effect that was inhibited by TSA (Fig. 4K,L,R). In both pBabe and Myc expressing keratinocytes, H4monoK20 increased on treatment with TSA (Fig. 4M–Q).

**Figure 5:**
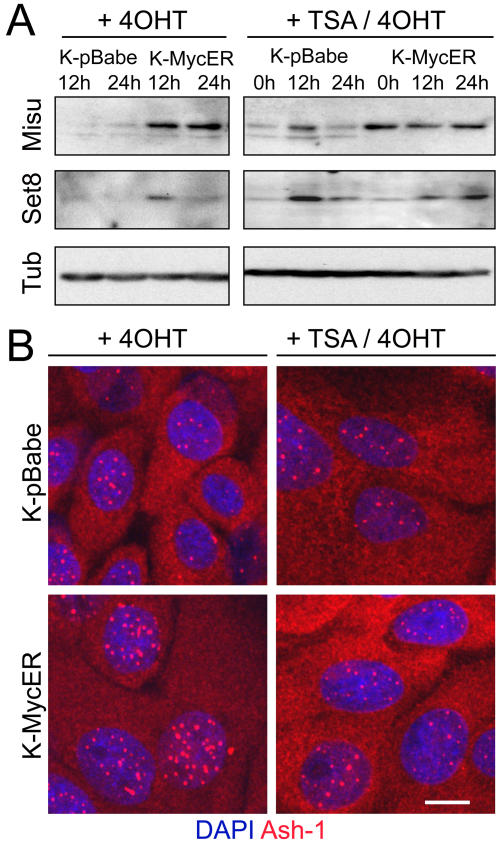
Expression of histone methyl transferases in response to Myc and TSA. (A) Western blots of keratinocytes infected with empty vector (K-pBabe) or MycER (K-MycER) and treated with 4OHT±TSA for the number of hours indicated. Blots were probed with antibodies to the proteins indicated. Tubulin (Tub) was used as a loading control. (B) Immunostaining for Ash-1 (red) with DAPI counterstain (blue). Scale bar: 10 µm.

Replacement of H4monoK20 with H4diK20 in response to Myc activation should be accompanied by activation of specific histone methyltransferases. Set8 is an H4K20 specific mono-methylase [29], [30], while H4K20 di-methylation is dependent on the histone methyltransferase Ash-1 [31]. Western blotting showed that expression of Set8 was transiently up-regulated within 12 hours of activating Myc in keratinocytes (Fig. 5A; left hand panel). Induction of the RNA methyltransferase Misu (NSun2), a direct down-stream target of Myc [16], served as a positive control for Myc activation in the Western blots (Fig. 5A; left hand panel).

TSA treatment had different effects on control keratinocytes and keratinocytes with activated Myc. In control cells transduced with pBabe, TSA caused a transient upregulation of Set8 (Fig. 5A; right hand panel), which may reflect an initial increase in S phase cells (detected as elevated Misu expression), followed by arrest in G2+M of the cell cycle [16]. In TSA treated cells with activated Myc, Set8 was highly expressed at 12 and 24 h (Fig. 5A; right hand panel). The effect of activation of Myc on Ash-1 was to stimulate nuclear accumulation, a process that was inhibited when the cells were treated with TSA (Fig. 5B).

### Inhibition of HDACs has similar effects on the epidermal stem cell compartment to activation of Myc

**Figure 6:**
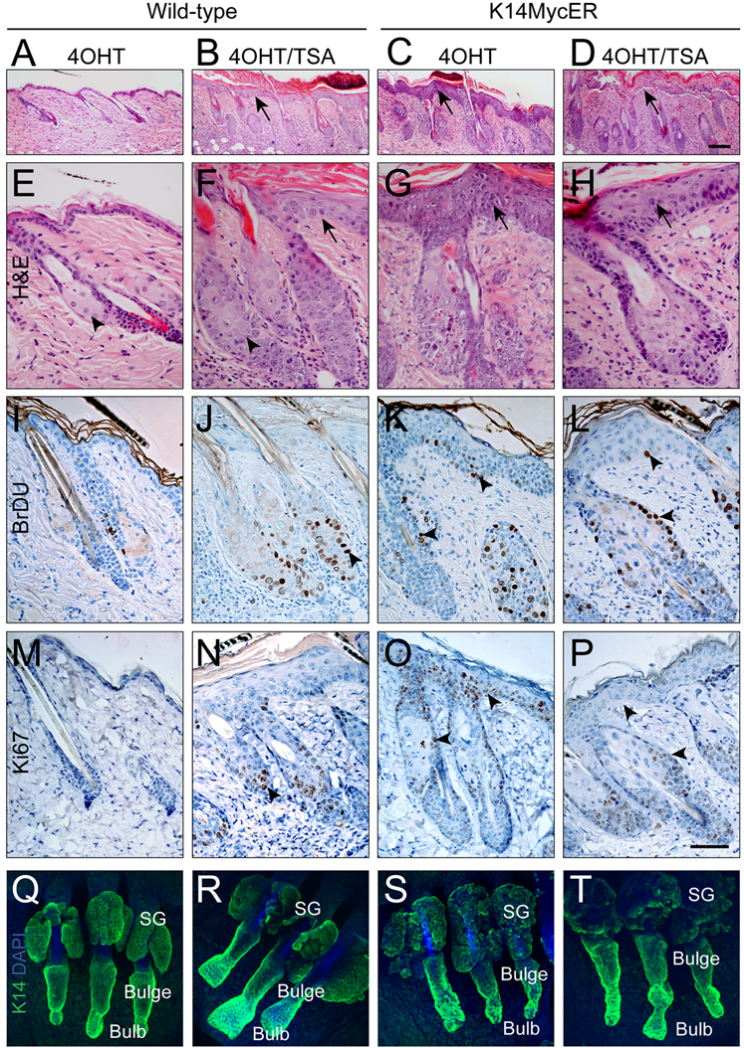
TSA treatment induces similar changes in epidermal proliferation and differentiation to activation of Myc, and exacerbates the effects of Myc. Wild-type (A, B, E, F, I, J, M, N, Q, R) and K14MycER transgenic (C, D, G, H, K, L, O, P, S, T) mice were treated with 4OHT alone (A, C, E, G, I, K, M, O, Q, S) or in combination with TSA (B, D, F, H, J, L, N, P, R, T). 4OHT treatment of wild-type epidermis served as a negative control. A–P are sections of back skin; Q–T are whole mounts of tail skin. Sections were stained with haematoxylin and eosin (A–H), anti-BrdU (brown, I–L) or anti Ki67 (brown, M–P). Whole mounts were stained for keratin 14 (green) with DAPI counterstain (blue). Arrows in B, C, F, G, H show thickening of interfollicular epidermis. Arrow in D shows detachment of epidermis from dermis. Arrowhead in F shows enlarged sebaceous gland. Arrowheads in J–L, N–P show different numbers of proliferating cells. SG: sebaceous glands. Scale bars: 100 µm

Our results show that acetylation of H4 and mono-methylation of H4 at lysine 20 are low in epidermal stem cells, and that activation of Myc or treatment with TSA increases both modifications. If the modifications contribute to the mechanism of Myc-induced exit from the epidermal stem cell compartment [11], [23], then TSA treatment of wild-type mouse epidermis should result in a similar phenotype to Myc activation. Indeed we found that both TSA treatment of wild-type epidermis and 4OHT treatment of K14MycER epidermis led to a marked increase in the number of cell layers in the interfollicular epidermis (Fig. 6A–C and E–G; arrows), enlargement of the sebaceous glands (Fig. 6E–G; arrowheads and Q–S) and expansion of the hair follicle bulb (Fig. 6Q,R). TSA treatment, like Myc activation, led to an increase in proliferation, as measured by Ki67 labelling and incorporation of a 1 hour pulse of BrDU into S phase cells (Fig. 6I–K, M–O; arrowheads).

Activation of Myc in the presence of TSA exacerbated the phenotype of K14MycER epidermis (Fig. 6D,H,L,P,T). There was massive accumulation of cornified cells in the IFE (Fig. 6D); in some areas no viable cell layers remained and the epidermis began to detach from the underlying dermis (Fig. 6D; arrow). Both TSA and Myc cause arrest of cultured keratinocytes in G2/M-phase of the cell cycle, prior to initiation of terminal differentiation [16], [23]. Consistent with these findings, the number of proliferative cells was lower in K14MycER epidermis treated with 4OHT and TSA (Fig. 6L,P) than with 4OHT alone (Fig. 6K,O). In addition to the effects on the interfollicular epidermis, the combination of TSA treatment and Myc activation resulted in further increases in the number of differentiated sebocytes (Fig. 6H; arrowhead and 6T) and aberrant hair follicle morphology (Fig. 6T).

We conclude that Myc-induced chromatin modifications play a major role in Myc-induced exit from the stem cell compartment and differentiation into interfollicular epidermis and sebocytes.

## Discussion

**Figure 7:**
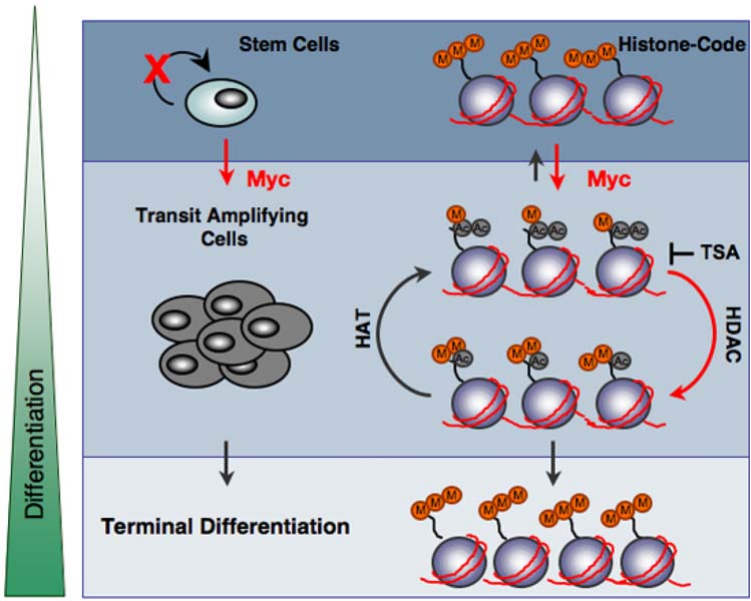
Model of histone modifications in different epidermal compartments and effect of Myc activation. Red arrows indicate events stimulated by Myc. Stem cells and terminally differentiated cells are shown as having high levels of histone tri-methylation and low levels of histone acetylation, mono- and di-methylation. Myc increases global H4 acetylation and transient mono-methylation of H4K20, which is replaced di-methylation of H4K20, H3K4 and H3K9 at the expense of tri-methylation. HAT: histone acetyl transferase; HDAC: histone deacetyl transferase complex.

We have demonstrated that quiescent epidermal stem cells in both the interfollicular epidermis and the hair follicle are characterised by high levels of tri-methylated H3K9 and H4K20 and low levels of H4 acetylation and H4K20 mono-methylation (Fig. 7). We observed that whereas bulge LRC had high levels of di-methylated H3K9 and H3K4, LRC in the IFE did not. While we do not have a functional explanation for this difference, it is consistent with reports of differences in the properties of stem cells in the bulge and IFE and may reflect differences in sensitivity to intrinsic and extrinsic factors [32], [33], [34].

In other cell types epigenetic plasticity is associated with under-representation of repressive histone marks [6], [7]. Epidermal stem cells, however, had high levels of histone H3triK9 and H4triK20, indicating that large sets of genes are repressed by Polycomb group (PcG) complexes. In embryonic stem cells, PcG proteins regulate stem cell pluripotency by maintaining repression of alternative lineage genes that are necessary for differentiation of stem or progenitor cells into various tissue types [35]. A similar situation might exist in adult epidermis. Although epidermal stem cells have the capacity to differentiate along multiple lineages, in undamaged, steady-state epidermis they appear to be specified to differentiate along only one or a small number of lineages [10]. This would be consistent with our observation that the terminally differentiated cells in the epidermis are, like stem cells, rich in H3K9 and H4K20 trimethylation. It also raises the interesting possibility that transit amplifying cells, being low in repressive histone marks (Fig. 7), are more susceptible to lineage reprogramming than stem cells [10].

In response to Myc activation epidermal stem cells enter the transit amplifying compartment, leading to a transient increase in proliferation followed by exit from the cell cycle and initiation of differentiation [11], [12], [23]. Exit from the stem cell niche is correlated with global histone H4 hyperacetylation, and transient histone H4 monomethylation at lysine 20 followed by stable dimethylation (Fig. 7). The increase in global histone H4 acetylation in response to Myc is in line with previous observations. Histone acetylation is known to be enhanced by Myc and is, at least in part, mediated by the direct up-regulation of GCN5 by Myc [19], [36]. Deletion of N-*myc* in neural progenitor cells leads to chromatin changes associated with chromatin inactivation, such as histone H3 and H4 hypoacetylation and increased di- and tri-methylation at histone H3 at lysine 9 [19].

Myc has the highest affinity for target genes in open, preacetylated chromatin and further enhances histone acetylation [36]. However, Myc binding and acetylation do not systematically correlate with gene activation [36], [37]. H4 acetylation is also reported to distinguish coding regions from heterochromatin independent of transcriptional state [38]. Myc-induced hyperacetylation may therefore represent a global epigenetic mark for coding regions that facilitates the binding of transcription factors required for subsequent positive or negative regulation of gene expression.

Although hyperacetylation of histone H4 has been found to correlate with mono-methylation at lysine 20 in promoter and coding regions of active genes [26], Myc-induced hyperacetylation correlated with only a transient increase in mono-methylation and with stable accumulation of nuclei with high levels of di-methylated H4K20, H3K4 and H3K9 (Fig. 7). In contrast to our observation that di-methylation increased in response to Myc, high levels of H3diK9 and H3triK9 are also found in N-myc null neural progenitors [19]. These results might indicate that di-methylation, at least at H3K9, is not directly regulated by Myc or involves additional factors. Nevertheless, the increased di-methylation in response to Myc in skin correlated with expression of the corresponding histone methyltransferases Set8 and Ash-1 [29], [30].

Our finding that di-methylation at different histones was partitioned together might be a general mechanism of regulating chromatin structures. Mono-methylated H4 at lysine 20 and H3 lysine 9 are partitioned together, as are the di-methylated forms, whereas mono- and di-methylated forms are virtually mutually exclusive [39]. In addition, chromatin marks that are associated with highly active promoters partition together and allow high affinity binding of transcription factors like Myc [40].

Histone acetylation can either be induced by Myc activation or by inhibiting deacetylation via TSA. TSA treatment also led to a major increase in the levels of H4monoK20 in the epidermis. The strikingly similar effects of Myc and TSA in stimulating proliferation followed by differentiation strongly suggest that histone hyperacetylation is part of the mechanism by which keratinocytes initiate terminal differentiation [11], [41].

HDAC inhibitors are currently in clinical trials for various types of cancer. These drugs can potentially reverse the resistance of tumour cells to traditional chemotherapeutic drugs [42]. Our observations suggest that, in addition, HDAC inhibitors could be beneficial because they promote differentiation. However, by mobilizing stem cells to leave the niche the drugs could also have the undesirable effect of compromising epithelial homeostasis in healthy tissues.

Our data suggest that activation of Myc induces a widespread change in chromatin state that is permissive for transcription factor binding. In response to activated Myc chromatin modifications that are associated with both chromatin silencing and gene activation were increased. This indicates that Myc induces an intermediate state of facultative heterochromatin and might delay the formation of obligate heterochromatin. This would explain why the epidermis of K14MycER transgenic mice displays both hyperproliferation and increased terminal differentiation. It would also explain how a single transcription factor can exert such broad pro-differentiative and pro-tumorigenic effects.

## Materials and Methods

### Transgenic mice and treatment

K14MycER transgenic mice (2184C.1) [11] and nontransgenic littermates (wild-type controls) received topical applications of 4OHT (Sigma, 1 mg per mouse per day) or trichostatin A (TSA) (Sigma, 1mg per kg per day) to a shaved area of dorsal skin. 4OHT and TSA were dissolved in ethanol. When mice were treated with both 4OHT and TSA, the animals received 4OHT or TSA on alternate days over the treatment period of 6 days. Mouse breeding and experimental protocols were subject to Institutional ethical approval and were performed under the terms of a U.K. Government Home Office licence.

### BrdU labelling

To generate label-retaining cells (LRC), 10-day-old mice were injected with 50 mg/kg (body weight) 5-bromo-2′-deoxyuridine (BrdU; 20 µl of 12.5 mg/ml BrdU) every 12 hours for a total of four injections, as described previously [22]. In some experiments mice were injected with BrDU one hour prior to sacrifice, in order to label S phase cells.

### Tissue preparation

Frozen sections (5–7 µm) of mouse dorsal and tail skin were fixed for 10 minutes in 4% paraformaldehyde prior to labelling. Wholemounts of mouse tail skin [22] and adult human skin [9] were prepared as described previously. Human skin was obtained as surgical waste from mastectomy operations with appropriate ethical approval.

### Immunostaining and image analysis

Immunostaining was performed as described previously [15], [16]. Antibodies used were: H3diK4 (clone RR302; Upstate), H3diK9 (kindly provided by T. Jenuwein), H3triK9 (kindly provided by T. Jenuwein), H4Ac (anti-acetyl-Histone H4; Upstate), ß1 integrin (P5D2) [43], H4monoK20 (ab9051; Abcam), H4diK20 (ab14092, Abcam), H4triK20 (ab9053; Abcam), Set8 (PR/SET07; Abgent), Ash-1 (ab4477; Abcam), tubulin (Sigma), Misu (NSun2) (EF1) [16], keratin 14 (MK14, Babco), keratin 10 (MK10, Covance), Ki67 (Novacastra), BrDU (Becton Dickinson) and Myc (N262, Santa Cruz).

Images were acquired and expression levels were estimated using a Zeiss 510 confocal microscope. Approximately 30 optical sections of each epidermal sheet were captured with an increment of 1 µm. Scans are presented as z-projections, scanned from the dermal side towards the epidermal surface. The number of LRC was counted per hair follicle bulge and per optical field in the interfollicular epidermis (Zeiss 20/NA 0.75).

### Human keratinocyte culture, retroviral infection and Western blotting

Primary human keratinocytes were isolated from neonatal foreskin and cultured in the presence of a feeder layer of J2-3T3 cells in FAD medium (1 part Ham's F12 medium, 3 parts Dulbecco's modified Eagle's medium (DMEM), 1.8×10^−4^ M adenine) supplemented with 10% fetal calf serum (FCS) and a cocktail of 0.5 µg / ml hydrocortisone, 5 µg/ml insulin, 10^−10^ M cholera toxin and 10 ng/ml epidermal growth factor (EGF) as described previously [23]. J2-3T3 cells were cultured in DMEM containing 10% donor calf serum.

Keratinocytes were infected with the following retroviral vectors: pBabe puro (empty vector) [44] and pBabeMycER [23]. Keratinocytes were infected by co-culture with retroviral producer cells as described previously and used within one or two passages after infection [23]. Activation of the steroid-inducible constructs was performed by adding 200 nM 4OHT (Sigma) to the culture medium.

Keratinocytes were solubilised in RIPA buffer, resolved by SDS-polyacrylamide gel electrophoresis and subjected to Western blotting as described previously [15].

## Supporting Information

Figure S1 Quantitation of immunofluorescence labelling in epidermal whole mounts. The fluorescence signal (y axes) along lines (white arrows; x axes) crossing representative regions of the bulge or interfollicular epidermis (IFE) double labelled for LRC (red) and histone modifications (green) is shown. Peaks of fluorescence correspond to nuclei. When the peaks of red and green coincide an LRC has a high level of the histone modification (asterisks), but when the red peak corresponds to a low level of green the LRC has a low level of the modification (circle). This is the basis for the histograms in Figures 2 and 3.
(5.41 MB TIF)
