# Supplementary figures and images for: Epidermal Stem Cells Are Defined by Global Histone Modifications that Are Altered by Myc-Induced Differentiation
Source: PLoS One. 2007 Aug 22;2(8):e763. doi: 10.1371/journal.pone.0000763 (PMC1945016; doi:10.1371/journal.pone.0000763)

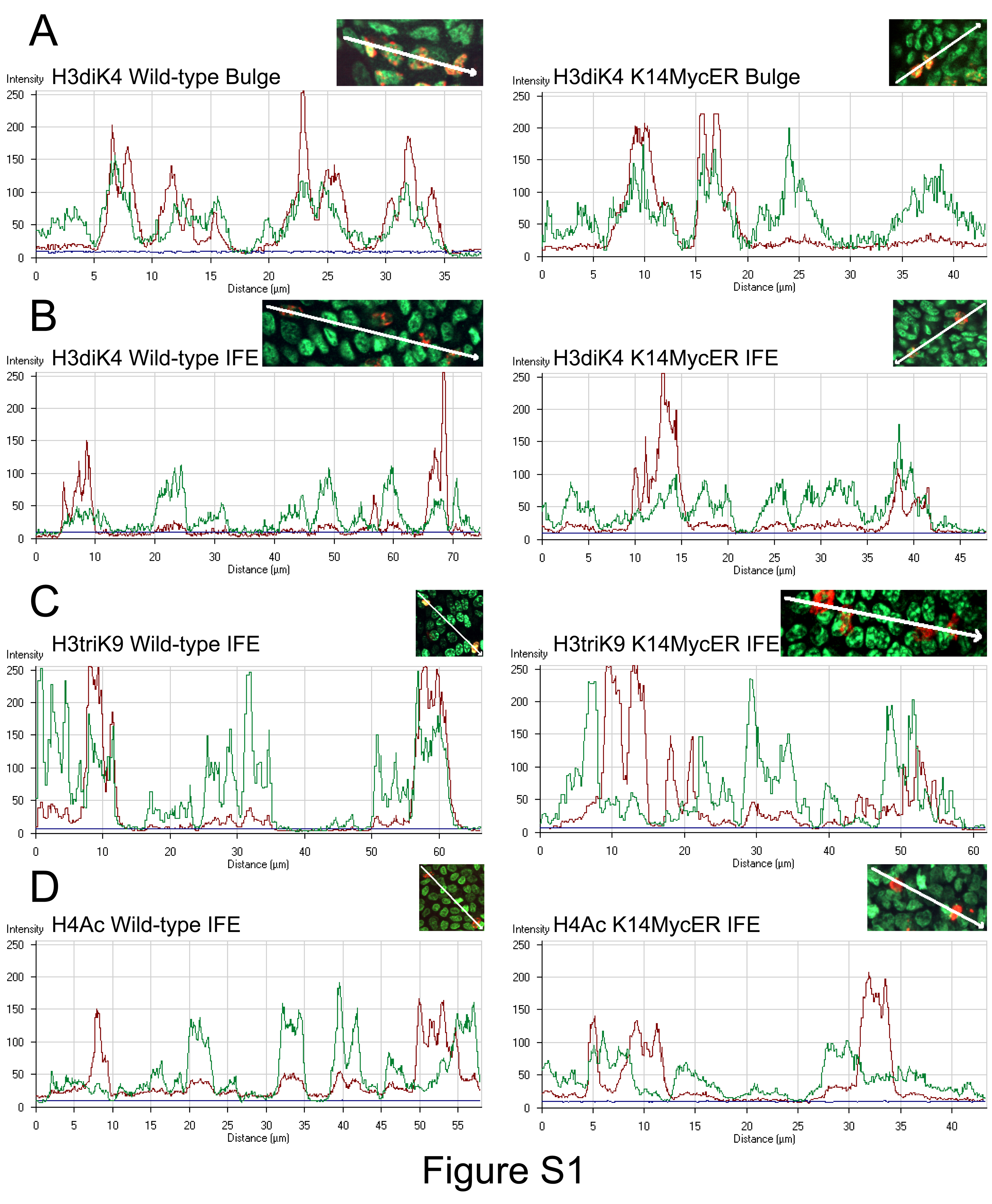

Supplement: Figure S1 — Quantitation of immunofluorescence labelling in epidermal whole mounts. The fluorescence signal (y axes) along lines (white arrows; x axes) crossing representative regions of the bulge or interfollicular epidermis (IFE) double labelled for LRC (red) and histone modifications (green) is shown. Peaks of fluorescence correspond to nuclei. When the peaks of red and green coincide an LRC has a high level of the histone modification (asterisks), but when the red peak corresponds to a low level of green the LRC has a low level of the modification (circle). This is the basis for the histograms in Figures 2 and 3. (5.41 MB TIF) [file pone.0000763.s001.tif]
